# Functional Outcomes, Survival Rate, and Complications of Patellofemoral Arthroplasty: Mid-Term Results From Independent Center

**DOI:** 10.7759/cureus.31945

**Published:** 2022-11-27

**Authors:** Nuthan Jagadeesh, Rafael Sales-Fernández, Srinath Pammi, Ankur Kariya

**Affiliations:** 1 Trauma and Orthopaedics, Vydehi Institute of Medical Sciences and Research Centre, Bengaluru, IND; 2 Orthopaedics and Trauma, Wrightington Wigan and Leigh NHS Foundation Trust, Wigan, GBR

**Keywords:** isolated patellofemoral arthritis, survival rates following patellofemoral replacement, eq-5d, oxford knee score, patellofemoral arthroplasty

## Abstract

Background

Patellofemoral arthroplasty (PFA) is indicated for isolated patellofemoral arthritis. PFA is a less invasive, bone-preserving procedure with faster recovery when compared to total knee replacement. We aim to present the functional outcome, survival rate, and complications of PFA from our center.

Methods

A total of 45 consecutive PFA performed (mean age 58.02 ± 9.2 years; 34 females and 11 males; Avon prosthesis) during the study period was included. Prosthesis survivorship was measured using revision as the endpoint, and functional outcome was assessed using Oxford knee scores (OKSs), EQ-5D, and satisfaction scores. Postoperative complications, the number of revisions, and the reasons causing them were collected.

Results

The mean follow-up was 48.7 (range 16-66) months. As compared to preoperative scores, OKS and Eq5d showed significant improvements (p<0.001). The satisfaction scores indicated that 28 patients (62%) were very satisfied, 10 (26.7%) were satisfied, and seven (15.5%) were dissatisfied. Four patients (8.9%) underwent revision surgery with a total knee replacement. Out of four patients requiring revision, two had progressive arthritis of the medial compartment, one had aseptic loosening of the implants, and one had trauma. Five patients showed persistent anteromedial knee pain (12.2%); one patient had maltracking requiring lateral release; one patient developed stiffness with knee flexion less than 60 degrees requiring manipulation under anesthesia.

Conclusion

Our results indicated that good or excellent functional outcomes can be expected in >80% of patients with survivorship of 91.1% at mid-term. Careful patient selection and counseling should be done informing of the risk of dissatisfaction/persistent pain and revision surgery in the mid and long term. Long-term outcomes and factors determining good outcomes need to be evaluated in future research.

## Introduction

Approximately 10% to 24% of patients suffering from knee osteoarthritis (OA) have isolated patellofemoral OA [1-3]. The indication for patellofemoral replacement is patients with isolated patellofemoral arthritis, incapacitating pain, functional limitations, and who have not responded to conservative management [4,5]. Arthroscopic debridement with or without lateral release, chondroplasty, unloading procedures with osteotomies of the tibial tuberosity, patellectomy, total knee arthroplasty (TKA), and patellofemoral arthroplasty (PFA) have all been used to treat isolated patellofemoral OA [6,7].

The initial results of PFA gave a high revision rate of 35.3% although the patellofemoral pain was alleviated in the short-term results [8]. It was therefore common practice for surgeons to recommend TKA over PFA when treating isolated patellofemoral osteoarthritis [9,10]. However, newer implant designs along with a better patient selection have improved outcomes. Recent systematic reviews recorded 92% overall survival rates for first- and second-generation PFA implants at five years, 83% at 10 years, 75% at 15 years, and 67% at 20 years [11]. The advantages of PFA are that it is a less invasive, bone-preserving procedure sparing both cruciate and tibiofemoral cartilage compared to TKA. In younger patients, PFA may act as a delaying procedure as a revision to TKA after PFA is less difficult than revision to TKA [12]. Moreover, patients with PFA have a shorter hospital stay and lesser blood loss which leads to faster recovery with excellent improvement in their functional outcomes [12,13].

Complications of the PFA are similar to those of the TKA, such as persistent pain, polyethylene wear, stiffness, loosening, etc. [14]. Among the early complications more common to PFA are patellar maltracking, clicking or snapping of the patella, anterior knee pain, and patellar fractures [15]. Better anticipation and understanding of the complications can help to minimize them. Based on the experience of an independent center, we present functional outcomes, survival rates, and complications associated with PFA.

## Materials and methods

This retrospective study involved 45 consecutive patellofemoral arthroplasties in a single tertiary care center between January 2015 and December 2020. All patients included in the study underwent a patellofemoral replacement for Grade 3/4 isolated patellofemoral arthritis (Iwano classification) and had a minimum follow-up of one year or more were included. Those patients not available for follow-up for a minimum of one year were excluded. The study was registered in the Audit and Research department of the trust and ethical approval was obtained.

All patients had a weight-bearing anteroposterior, lateral, and skyline radiograph of the affected knee preoperatively. Preoperative diagnosis of isolated severe patellofemoral arthritis was established based on the history of disabling anterior knee pain and radiological evidence of grade three or four patellofemoral arthritis using Iwano classification and arthroscopic findings wherever applicable. MRI of the knee was done to establish the diagnosis as well as to know the status of cartilage at the tibiofemoral joint wherever necessary. Other preoperative parameters like age, sex, grade of tibiofemoral arthritis, and previous surgeries were collected.

**Figure 1:**
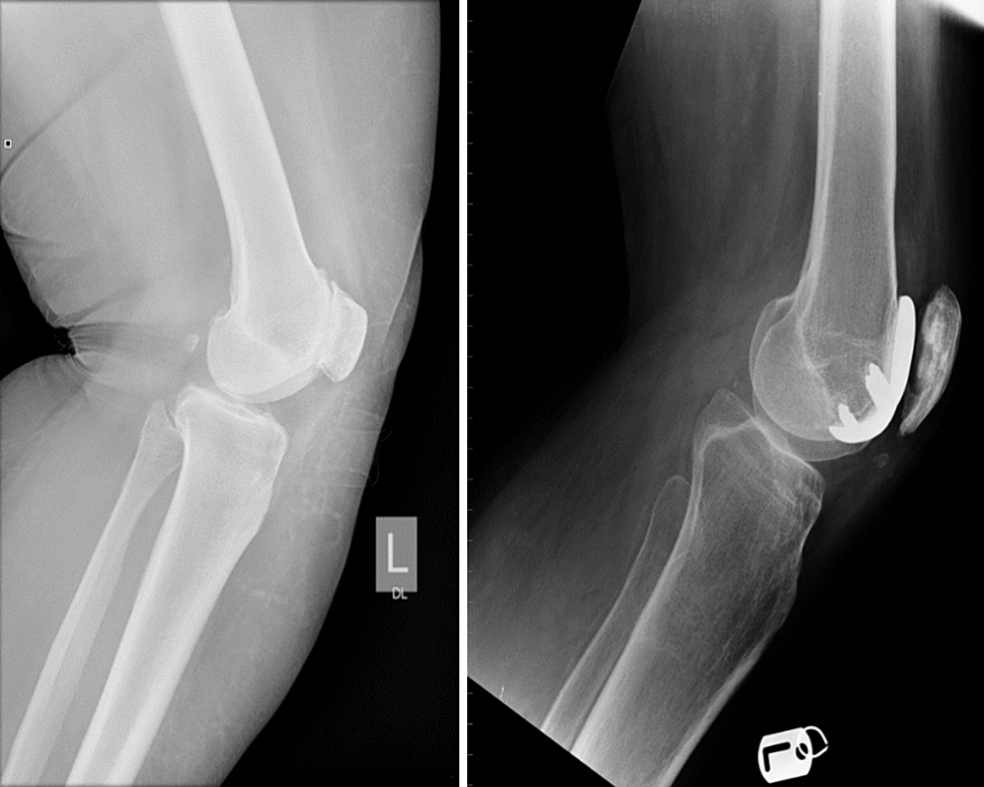
Preoperative and postoperative radiographs of the lateral view of the knee of a patient undergoing patellofemoral arthroplasty for isolated patellofemoral arthritis

All operations were performed by five trained experienced orthopedic consultants working in tertiary elective centers in the United Kingdom. The medial parapatellar approach was used for the exposure under the tourniquet. The Avon second-generation implant (Stryker Orthopaedics) with cement was used in all the cases (Figure 1). In addition, two patients underwent tibial tubercle transfer for patellar alta, one patient underwent lateral release to manage patellar tracking. All patients were made to mobilize full weight bearing the next day and were discharged when deemed fit. No patient required a blood transfusion. All patients received one dose of antibiotic (cefuroxime) at the time of induction and two further doses postoperatively.

After the preoperative assessment, patients were seen in a research clinic six weeks after surgery, six months later, and annually thereafter by an advanced physiotherapist practitioner, and the operating surgeon was involved if there are concerns raised. Prosthesis survivorship was measured using revision as the endpoint, and functional outcome was assessed using revised Oxford knee scores (OKSs) and EQ-5D scores. The audit and research department of the trust obtained patient-related outcome measures (PROMs), such as the OKS and the EQ-5D, preoperatively and during postoperative follow-ups. PROMs of the patients who were missed by the department were collected by telephone consultation by a research fellow not involved in the surgery. We could not obtain PROMs for three patients who were not available for telephonic consultation. Patient satisfaction score (very satisfied, satisfied, unsure, dissatisfied, and very dissatisfied) was collected from the clinic letters. PROMs scores were available for 42 patients. The mean follow-up at the latest follow-up appointment was 48.7 months (range 16-66 months). Postoperative clinic letters and case notes were reviewed to determine complications, the number of revisions, and the reasons causing them wherever documented.

Statistical analysis

Data were analyzed using SPSS software version 22 (IBM Corp., Armonk, NY). Mean and standard deviation was calculated for continuous data and percentage for categorical data. Paired t-test was used as a test of significance for comparing pre- and post-operative scores of OKS and EQ5D scores. A p-value of <0.05 was considered significant.

## Results

Forty-five patients who underwent PFA had a mean follow-up period of 48.7 (range 16-66) months. The mean age was 58.02 (± 9.2) years. 44.4% of patients had an age greater than 60 years. More than 75% of the patients were females. The mean BMI of the patients was 30.57 (± 4.27). Most patients (80%) had grade 0-1 whereas 20% of them had grade 2 tibiofemoral arthritis of Kellgren Lauren Classification (Table 1).

**Table 1:** Demographic variables and grading of patellofemoral and tibiofemoral arthritis

Parameters	Mean (± SD)	Subgroups	Frequency	Percent
Age	58.02 (± 9.2yrs)	41 - 50 yrs	12	26.7
		51 - 60 yrs	13	28.9
		> 60 years	20	44.4
		Sex	NA	Female	34	75.6
Male	11			24.4
BMI	30.57 (± 4.27)	NA	NA	NA
KL classification - Grade of tibiofemoral arthritis	NA	0	8	17.8
		1	28	62.2
		2	9	20
Patellofemoral arthritis- Iwano Classification	NA	3.0	1	2.2
		4.0	44	97.8

Postoperative PROMs (OKS and Eq5d) were available for 42 out of 45 patients. As compared to preoperative scores, OKS and Eq5d showed significant improvements (p<0.001) (Table 2). Twenty-two patients had excellent outcomes (OKS 40-49), 11 patients had good outcomes (OKS 30-39) and nine patients had moderate outcomes (OKS 20-29). None of the patients had poor outcomes (OKS <19). The satisfaction scores indicated that 28 patients (62%) were very satisfied, 10 (26.7%) were satisfied, and seven (15.5%) were dissatisfied. There were no very dissatisfied patients. Three patients whose PROMs were not documented were very satisfied with the surgery as per clinic letters with no complications.

**Table 2:** Preoperative and postoperative functional outcomes score using OKS and EQ-5D

Patient-related outcome measures	Preop (Mean ± SD)	Postop (Mean ± SD)	P-value
OKS	19.5±4.2	37.2±7.7	<0.001 *
EQ-5D	0.62±0.9	0.85±1.4	<0.001 *

Four patients (8.9%) underwent revision surgery with a total knee replacement. Out of four patients requiring revision, two had progressive arthritis of the medial compartment, one had aseptic loosening of the implants, and one had trauma requiring revision. Four patients had revisions at 2, 3.9, 4.2, and 4.6 years, respectively, after the primary surgery. Five patients showed persistent anteromedial knee pain (12.2%); one patient had maltracking requiring lateral release; one patient developed stiffness with knee flexion less than 60 degrees requiring manipulation under anesthesia. Two patients underwent arthroscopic excision of fibrofatty tissue (Table 3).

**Table 3:** Postoperative complications

Complications	Frequency	Percent
Clicking sound	2	4.4
Recurrent effusion	1	2.2
Loosening	1	2.2
Maltracking	1	2.2
Anteromedial pain	6	13.3
Stiffness	1	2.2
No complications	35	77.8

## Discussion

PFA was performed for the first time in the year 1955 by Mckeever to avoid patellectomy in patients with isolated patellofemoral arthritis [15,16]. PFA is indicated for isolated patellofemoral arthritis in whom conservative nonoperative measures are exhausted. The surgery involves resurfacing the patella and trochlea sparing the tibiofemoral joint, both cruciate, and menisci leaving a good bone stock for revision surgery. Historically, good short-term results were seen with the surgery but there are concerns raised about the progression of tibiofemoral arthritis requiring revision to total knee replacement [17,18]. Newer designs have improved the outcome, but concerns do remain [19,20]. Appropriate patient selection may be the key to getting better results. We aimed to assess the functional outcome, survivorship, and complications associated with PFA.

The mean age of patients involved in the study was 58.02 (± 9.2 years) (range 43-72 years) which was significantly lesser compared to the average age of 72 years in total knee replacements [21]. This is reflected by the fact that most patients are relatively young and there is a need to preserve bone stock for future revision surgeries. Most of the patients in our study were females (75%). A similar distribution is found in the registry as well as several studies previously published [4-6]. Women are 2.3 times more likely to have patellofemoral pain syndrome and develop subsequent patellofemoral arthritis [22].

Starks et al., in their study of 37 patellofemoral replacements, showed excellent early functional outcomes with median OKS of 39; a median Melbourne Knee score of 28 in postoperative follow-up, and only one patient reported unsatisfactory outcome [23]. Akhbari et al. in their mid-term functional results also showed good results with mean OKS of 31.8 at five years postoperatively and these were significantly better when compared to the preoperative scores (p<0.001) [24]. Abeysekara et al. also concluded that PFA significantly improved knee function with significant improvement in OKS [25]. Our results were similar to the above-published results with significantly improved OKS and Eq5d scores postoperatively compared to preoperative scores. The OKS score improved from 19.5 ±4.2 preoperatively to 37.2±7.7 postoperatively with >80% of patients having good to excellent results with OKS > 30. Similarly, Eq5d improved from 0.62±0.9 preoperatively to 0.85±1.4 postoperatively (p<0.001). Moreover, the majority of the patients were very satisfied with the outcome of the surgery.

According to the annual report 2022 of the national joint registry of England and Wales, the PFA still accounts for only 1.1% of all knee replacements with Avon remaining the commonest implant used [21]. The cumulative revision rates for three years and five years are 5.56% and 9.21%, respectively [21]. Van der List et al. in their systematic review of 39 studies calculated the survivorship of PFA was 91.7% at five years (10). According to the authors, tibiofemoral OA progression (38%), pain (16%), aseptic loosening (14%), and patellar maltracking (10%) were the leading causes of failure. Early failures (within five years) were mostly due to pain (31%), whereas late failures (46%) were more likely to be caused by OA progression. William et al. in their recent study of 56 patients had survivorship of 95% with five revisions [26]. Two of the five revisions were related to the progression of tibiofemoral arthritis. In our study, the revision rate was 8.9%; thus, giving the survivorship of 91.1%. Four patients had revisions at two, 3.9, 4.2, and 4.6 years, respectively. Among these, two patients had a revision of progressive tibiofemoral disease whereas one had mal-tracking and subsequent loosening of the implant. One patient had a revision due to a traumatic knee injury.

Four cases (8.9%) underwent re-operative procedures with implant retention for addressing complications. One patient had a lateral release for maltracking; one had manipulation under anesthesia for stiffness whereas two patients had arthroscopic excision of fibrofatty tissue to address soft tissue impingement. William et al. had re-operative procedures with implant retention in seven out of 56 patients (12%) in which three were lateral releases for maltracking of the patella. Another significant complication noted in our study was persistent anteromedial pain which was seen in 12.2% of the patients. This could be due to progressive arthritis in the tibiofemoral joint.

This study has several limitations. The retrospective nature study does not allow to draw of conclusions about preventable reasons for revisions or poor outcomes. The surgery was undertaken by more than one surgeon reduces the homogeneity of the study such as preoperative assessment and radiologic investigations available. The PROMs were not collected for three patients. Though these patients were followed up in the clinic as per protocol and were doing well without complications or had any revision surgeries, they had no PROMs collected and were not responding to telephonic consultations during data collection. The decision to revise was at the discretion of the operating surgeon. The outcome for some patients was unsatisfactory despite not having revision surgery, while the outcome of one patient with a traumatic knee injury before revision had been excellent. Despite these limitations, our results indicate that PFA provides a favorable functional outcome with acceptable survivorship in the mid-term period.

## Conclusions

Our results indicated that good or excellent functional outcomes can be expected in >80% of patients with survivorship of 91.1% at mid-term. Careful patient selection and counseling should be done informing of the risk of dissatisfaction/persistent pain and revision surgery in the mid and long term. Long-term outcomes and factors determining good outcomes need to be evaluated in future research.
